# P-1909. Social Connectedness in the COVID-19 Pandemic’s Aftermath: The Influence of Disease Status

**DOI:** 10.1093/ofid/ofae631.2070

**Published:** 2025-01-29

**Authors:** Christopher Kricorian

**Affiliations:** For Good Measure, Simi Valley, California

## Abstract

**Background:**

During the COVID-19 pandemic, maintaining social connectedness became difficult.As the pandemic receded, these challenges eased and allowed people to re-develop their social relationships. However, those with Long COVID (LC) may experience ongoing difficulties given the effects of their illness, but limited research has examined the association of LC with social connectedness.

**Methods:**

The US Census Bureau Household Pulse Survey data was used in this analysis. Data was collected online between January 9 - February 5, 2024, in English and Spanish. Sample size was N=68,544. Social connectedness metrics were compared between those reporting LC, COVID-19 infection that had resolved (Short COVID, or SC), and those who had not experienced COVID (No COVID, or NC). Data were analyzed using χ^2^ and between group contrast analysis.

**Results:**

When asked how often they feel lonely, those with LC were significantly more likely to say *always, usually,* or *sometimes* as compared to SC and NC respondents and less likely to say *rarely* or *never* (all comparisons to LC p< .05). In terms of receiving necessary social and emotional support, only 21.6% of those with LC said they *always* get necessary support, vs. 30.9% for SC and 30.0% for NC (both LC comparisons p< .05). LC respondents were most likely to say they got together with friends or relatives *less than once per week* (47.1%, vs. 35.3% SC and 43.1% NC, all comparisons p< .05). Those experiencing SC were most likely to say they texted with friends, family or neighbors *5+ times per week* (70.1%), followed by LC (65.1%), then NC (59.9%, all comparisons p< .05).

**Conclusion:**

People suffering from LC generally exhibited lower levels of social connectedness and had lower likelihood of obtaining necessary social and emotional support than those who experienced SC or NC. Additional research should be conducted to better understand the causal connections: are people with poorer social connections more likely to experience LC, does LC damage the ability to become or remain socially connected, or both? More research is needed to examine these questions and develop programs to help rebuild and retain social connections in the aftermath of the COVID-19 pandemic.

**Disclosures:**

All Authors: No reported disclosures

